# ﻿Two new species of the hyperdiverse geometrid moth genus *Eois* (Lepidoptera, Geometridae, Larentiinae) from Ecuador, with descriptions of early stages

**DOI:** 10.3897/zookeys.1192.111275

**Published:** 2024-02-21

**Authors:** Lydia M. Doan, James S. Miller, John W. Brown, Matthew L. Forister, Lee A. Dyer

**Affiliations:** 1 Department of Biology, Ecology, Evolution and Conservation of Biology, University of Nevada, Reno, NV 89557, USA; 2 Entomology Department, American Museum of Natural History, New York, NY, 10024, USA; 3 Entomology Department, National Museum of Natural History, Smithsonian Institution, Washington, DC, 20560, USA; 4 INABIO – Instituto Nacional de Biodiversidad, Quito, Ecuador; † Deceased

**Keywords:** COI, genitalia, morphology, Neotropics, *olivacea* species group, *Piper*, taxonomy

## Abstract

The hyperdiverse geometrid genus *Eois* Hübner, estimated to encompass more than 1,000 species, is among the most species-rich genera in all of Lepidoptera. While the genus has attracted considerable attention from ecologists and evolutionary biologists in recent decades, limited progress has been made on its alpha taxonomy. This contribution focuses on the Olivacea clade, whose monophyly has been recognized previously through molecular analyses. We attempt to define the clade from a morphological perspective and recognize the following species based on morphology and genomic data: *E.olivacea* (Felder & Rogenhofer); *E.pseudolivacea* Doan, **sp. nov.**; *E.auruda* (Dognin), **stat. rev.**; *E.beebei* (Fletcher, 1952), **stat. rev.**; *E.boliviensis* (Dognin), **stat. rev.**; and *E.parumsimii* Doan, **sp. nov.** Descriptions and illustrations of the immature stages of *E.pseudolivacea* reared from *Piper* (Piperaceae) in Ecuador are provided.

## ﻿Introduction

Larentiinae is the second largest subfamily of the highly diverse and worldwide family Geometridae, commonly known as geometers, loopers, or inch worms owing to the unusual gait of the caterpillars. Larentiines are primarily denizens of temperate regions, with more than 6,200 described species. However, the larentiine genus *Eois* Hübner is strictly tropical, and 83% of the described species are restricted to the Neotropics (Brehm et al. 2005; Strutzenberger et al. 2010, 2011, 2017; Brehm et al. 2011; Õunap et al. 2016). The genus is comprised of 267 formally described species: 220 in the Neotropics (Moraes et al. 2021a, b), 30 in Southeast Asia, and 17 in Africa (Herbulot 2000; De Prins and De Prins 2023). Remarkably, it is estimated that an additional 1,000 or more Neotropical species of *Eois* remain to be described (Brehm et al. 2011; Strutzenberger et al. 2017; Moraes et al. 2021a, b). If these estimates are correct, the genus is among the most species-rich in all of Lepidoptera. Based on Lepidoptera inventories in Central and South America, *Eois* appears to reach its greatest diversity in high elevation (higher than 2,000 m) habitats of the eastern Andes (Brehm et al. 2005; Brehm et al. 2011; Õunap et al. 2016).

Species of *Eois* are generally small, with wingspans of 12–20 mm. Wing shape and pattern are diverse, featuring ground colors of yellows, greens, or browns, some with finely reticulated networks of lines, some with spots or bands, and others nearly uniform in color (Moraes et al. 2021a). Larvae are mostly green, with brown, red, or black spots or bands; others are completely dark (Brehm et al. 2011: fig. 6; also see http://caterpillars.org). In some clades, larvae are elongate and transparent greenish, whereas in others they are stout and brightly colored; one species even appears to mimic bird-droppings (Brehm et al. 2011). The larvae exhibit a typical geometrid ground plan, with slender bodies, the absence of prolegs on abdominal segments 3–5, and slight modifications to the basic setal pattern found in other larentiines (McGuffin 1958).

The majority of *Eois* species were described between 1891 and 1920; but the first species was named by Hübner in 1818 and the most recent by Moraes in 2021 (Parsons et al. 1999; Moraes et al. 2021a, b). While progress on the taxonomy of the genus has been slow since the 1950s, during the past two decades *Eois* has experienced a resurgence in attention from ecologists and evolutionary biologists focused on the interactions of *Eois* immatures with their host plants and associated parasitoids (e.g., Dyer and Palmer 2004; Connahs et al. 2009; Brehm et al. 2011; Strutzenberger and Fiedler 2011; Strutzenberger et al. 2012; Seifert et al. 2015). *Eois* larvae are specialized feeders on shrubs and vines of Piperaceae (mostly *Piper*), and their diversification mirrors the substantial diversification of their larval hosts (Rodriguez-Castaneda et al. 2010; Jahner et al. 2017). Strutzenberger et al. (2010) indicate that *Eois* larvae also feed on Chlorantaceae, but out of more than 10,000 rearing records from Costa Rica, Ecuador, Brazil, Peru, and Argentina (Dyer et al. 2007; Forister et al. 2015; Salcido et al. 2022, and Janzen/Hallwachs databases, Dyer et al. databases), there are fewer than 300 records of this plant family.

A preliminary molecular phylogeny of the genus based on the mitochondrial gene cytochrome oxidase subunit 1 (COI; 1220 bp) and the nuclear gene elongation factor 1 alpha (Ef-1a; 1066 bp), evaluating 142 taxa, was presented by Strutzenberger et al. (2010), and this was followed by a checklist of the Neotropical species compiled by Brehm et al. (2011). A second analysis by Strutzenberger et al. (2017) using the same genes and a total of 221 *Eois* species, confirmed and reinforced their previous findings. The most recent molecular study of the genus by Moraes et al. (2021b) suggests that *Eois* potentially harbors an unparalleled array of cryptic diversity. Taken together, these molecular studies provide a preliminary phylogenetic framework for taxonomic progress at the species level, through the identification of many well-defined clades that can now be investigated based on their monophyly.

In each of the molecular studies, a well-defined “Olivacea Clade” was recognized that is rich in undescribed species from South America, primarily Ecuador (Strutzenberger et al. 2010, 2017; Moraes et al. 2021b), and this is supported by the large number of BINs represented in BOLD (Barcode of Life Database). Thus, we concentrate here on the Olivacea clade as a productive and diverse locus for the discovery and description of new species within the enormous undescribed diversity of the genus *Eois*. We utilize extensive morphological characters in this work, rather than molecular data, acknowledging that molecular identification has become an important part of modern taxonomy, sometimes in the form of a short section of mitochondrial DNA. The utilization of a single molecular marker (or “barcode”) has a number of issues (Taylor and Harris 2012, Mallo and Posada 2016) that are especially problematic in rapidly diversifying and under-documented lineages (Meyer and Paulay 2005). Genomic resolution, essential to the future of taxonomy, has been used in *Eois* (Jahner et al. 2017), and we expect that our work here helps lay the foundation for such studies moving forward. Also, the focus here is on describing two commonly reared species in Ecuador that have been and continue to be important in ecological and evolutionary studies (Dyer et al. 2007; Wilson et al. 2012; Forister et al. 2015; Glassmire et al. 2016; Jahner et al. 2017; Salcido et al. 2022; Sudta et al. 2022).

## ﻿Materials and methods

### ﻿Specimens examined

The bulk of the material used in this treatment is from two sources: the type collection of the National Museum of Natural History, Washington, D.C. (**USNM**), and an inventory of the caterpillars at Yanayacu Biological Station, Napo Province, Ecuador (Miller and Dyer 2009; Sudta et al. 2022). The Yanayacu site is located at 2200 m elevation in the Quijos Valley, Napo Province, in the Andes Mountains of northeastern Ecuador. The station lies just south of the equator (00°35.9'S, 77°53.4'W) in one of the world’s last remaining unexplored regions of high-elevation cloud forest. The survey has run continuously from 2001 to present.

At Yanayacu, caterpillars were discovered in the field primarily using visual searches. Larvae were taken to the laboratory where they were placed in plastic bags that were coded, imaged, tagged, and hung on clothes lines. Periodically, observational notes were taken on the larvae, and additional host material was added as needed. When an adult moth or butterfly, or sometimes its parasitoid, emerged, it was preserved and labeled. Each specimen received a unique voucher number in the form of a serial number (e.g., 15328), with time of year, elevation, latitude and longitude, host plant, and other data. In the text, we use “**r.f.**” (reared from) to denote larval host plants. Some specimens were preserved in alcohol and examined for setal patterns and other important larval characters such that a general description of shared characters among *Eois* larvae from this location is possible. Adults were collected at light traps at Yanayacu throughout the study period, and immatures and adults were collected at sites across the Neotropics (Forister et al. 2015; Salcido et al. 2022), and there was no overlap with the reared material from Yanayacu.

### ﻿Morphological data

We utilized a morphological species concept to delineate and describe the newly discovered species. Our approach focused on the examination of morphological characters, with an emphasis on the male and female genitalia. We used a matrix comprising 107 morphological characters to assess variation among individuals and delineate species (see Appendix 1). The data matrix included 37 binary and 70 multi-state characters: 16 external features (wings and other appendages), 58 male genitalia characters, and 33 female genitalia characters. The matrix was based on identified specimens in the collection of the University of Nevada, Reno (**UNR**); the Natural History Museum, London (**NHMUK**); the McGuire Center for Lepidoptera and Biodiversity, Florida Museum of Natural History, Gainesville, Florida (**AME**); and the National Museum of Natural History, Smithsonian Institution, Washington, DC (**USNM**; all USNM specimens are labelled with a unique USNM ENT barcode label, the numbers of which are given in the text).

In our results below, we also include redescriptions of three previously described species of the Olivacea Clade, which are based only on males. We present the first published illustrations of the adults and genitalia of *E.auruda* and *E.boliviensis*, and contemporary illustrations of *E.beebei*; those in the original publication of the latter are of poor quality.

Based on previously published data, the extensive BOLD database, and our own personal experience, species of *Eois* exhibit narrow geographic ranges, with none of the described species documented thus far beyond the limits of a single country. This distributional feature adds support to the morphological hypothesis that *E.olivacea* (described from Colombia), *E.boliviensis* (described from Bolivia), *E.beebei* (described from Venezuela), and *E.auruda* (described from Ecuador) are unlikely to be conspecific. Hence, these taxa are returned to species-level.

### ﻿Dissections and photography

Dissection methods followed those presented by Robinson (1976), except all parts were slide mounted using Euparal rather than Canada balsam. Initially, we attempted to evert the vesica, teasing it out with a 000 pin, but owing to the small size of the phallus, the process frequently inflicted more damage than the value of viewing features of the everted vesica. Also in the male genitalia, a membranous region surrounding the phallus typically supports a dense field of small spines. Although we attempted to leave the membrane in situ, more often it was detached with the phallus. For mated females, any spermatophores and associated material were removed.

Images of adults and genitalia were captured using a 65 mm lens attached to a Canon EOS 40D digital SLR camera (Canon U.S.A., Lake Success, NY) mounted on a Visionary Digital BK Lab System (Visionary Digital, Palmyra, VA). Multiple images were stacked using Helicon Focus software and subsequently enhanced using Adobe PhotoShop and GIMP 2.10 software. Plates were constructed in Adobe Photoshop CC 2020 (v. 21. 0).

### ﻿Terminology

Descriptions of morphology and wing maculation are based on the examination of specimens using a Zeiss Stemi 2000-C stereomicroscope with SCHOTT EasyLED ring-light illuminator. Forewing length was measured to the nearest 0.5 mm using an optical micrometer. Terms for genital structures and forewing pattern elements follow Holloway (1997) and Viidalepp (2011). However, an unusual structure in the male genitalia of members of several clades of *Eois* appears to lack a term. It is a membranous, flat, lateral flap of variable size and length attached to the sides of the tegumen and/or transtilla, which supports long, fine male scent scales. We suggest the term “lacina” for these structures and use that term throughout the manuscript. Viidalepp (2011) indicates that “The paired, heavily sclerotized, long and tapered projections from the posterior side of tegumen, which are the peculiarity of two species of the genus *Solitanea* Djakonov (the tribe Solitaneini Leraut) can be identified neither with socii nor with gnathos.” Although we are uncertain of the homology of lacina with the structures in *Solitanea*, they appear to occupy the same position in the male genitalia.

Abbreviations for morphological structures in the text are as follows: **Tg7** = tergum 7; **Tg8** = tergum 8; **St7** = sternum 7; **St8** = sternum 8; **PVP** = postvaginal plate; **DB** = ductus bursae; **DS** = ductus seminalis; **CB** = corpus bursae.

For the older type material (i.e., *E.auruda*, *E.boliviensis*, and *E.beebei*), we provide latitude, longitude, and elevation based on the locality data from the specimen label. Therefore, owing to the often imprecise nature of these label data, these parameters represent estimates only.

## ﻿Results

### ﻿The Olivacea clade

The Olivacea clade was first recognized by Strutzenberger et al. (2010) based on an analysis of 142 morphospecies of *Eois*, employing sequence data from two genes: COI (1220 bp) and Ef-1a (1060 bp). Sixteen morphospecies linked together to form the clade, all of which were assumed to be undescribed. The monophyly of the clade was subsequently confirmed by Strutzenberger et al. (2017) based on considerably broader taxon sampling (*n* = 221 morphospecies) but with the same genes, expanding the number of morphospecies in the clade to 23. A recent molecular study by Moraes et al. (2021a, b) found support for an *olivacea* species group, but not for the clade; however, their study was based entirely on the mitochondrial gene COI.

A preliminary genomic study of four species of *Eois* in the Olivacea Clade, all of which are undescribed, represented by 137 samples (Doan 2023), is the subject of ongoing work and a forthcoming publication. Similarly, a morphological analysis is in progress, based on a subset of *Eois* species (94 taxa and 107 morphological characters), and this will contribute to the delineation of the Olivacea Clade (Doan 2023).

Brehm et al. (2011: 1093) noted that all species belonging to the clade “have a green ground color and yellowish fringes…” They also commented that the caterpillars “show particularly contrasting patterns, including bright and dark spots dorsally…and pink spots laterally…in some species, whereas others exhibit merely some pale patches…” (Brehm et al. 2011: 1094–1095). They concluded that all species of the clade feed on species of *Piper* (Piperaceae). They further recognized that conspicuous morphological differences could be found even among closely related species within the group. For example, the male genitalia of some taxa possess numerous stout cornuti in the vesica, whereas others lack them altogether. Within the Olivacea Clade as defined by Strutzenberger et al. (2010, 2017), there appears to be three species complexes: an *E.olivacea* complex, an *E.goodmanii* complex, and an *E.muscosa* complex (although the last is represented by a single morphospecies). Although the first two species complexes were recognized as monophyletic by Moraes et al. (2021b), they were included as part of a larger clade, and *muscosa* was not included in their study. We focus our current efforts on the *E.olivacea* species complex. However, because diagnostic morphological characters to separate the *E.olivacea* species complex from the *E.goodmanii* species complex are yet to be discovered, one or more of the species treated herein may belong to the latter.

***Eoisolivacea* species complex**

**Remark.** As currently defined, the *E.olivacea* species complex includes *E.olivacea* (Felder and Rogenhofer 1875) (TL: Bogotá, Colombia; type in NHMUK), *E.beebei* (Fletcher, 1952) (TL: Rancho Grande near Maracay, Venezuela; type in NHMUK), *E.auruda* (Dognin, 1900) (TL: Loja, Ecuador; type in USNM), and *E.boliviensis* (Dognin, 1900) (TL: Bolivia, type in USNM), along with nearly a dozen undescribed morphospecies treated by Strutzenberger et al. (2017). BOLD (Barcode of Life Database) includes 27 BINs, mostly from Ecuador, with many fewer from Peru and Colombia, that likely represent species in the *olivacea* species complex. Herein, we describe two new species based nearly entirely on morphology of the adults. We also provide superficial descriptions and illustrations of the egg, larva, and pupa of *E.pseudolivacea*.

To minimize redundancy in the species descriptions, we first provide a general diagnosis and description of the *E.olivacea* species complex, which includes features shared by all the included taxa. In the individual species descriptions that follow, we include diagnoses and descriptions that include details of the features treated broadly in the general description of the complex.

**Figures 1–4.**
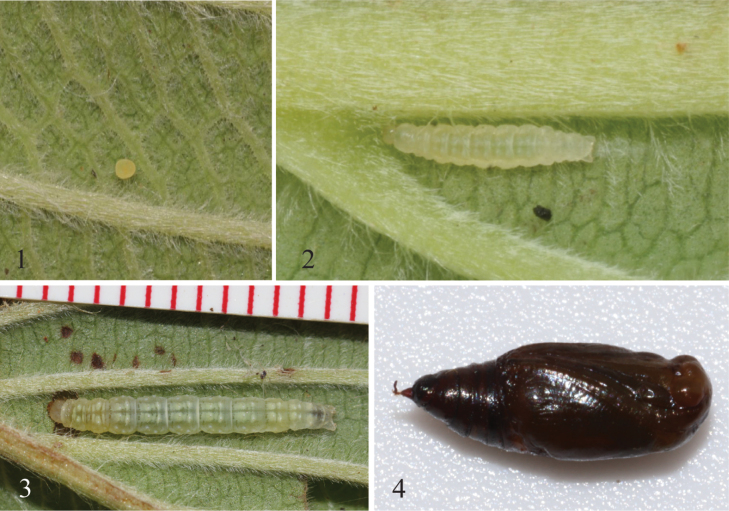
Early stages of *Eoispseudolivacea* from Ecuador **1** egg **2** second instar larva **3** fifth instar larva **4** pupa.

**Diagnosis.** Ovum (Fig. 1). The eggs are uniformly cream colored, oval, and flattened without sculpturing, and are deposited horizontally (i.e., not upright). They are laid singly on a leaf, but infrequently multiple eggs, as many as 12, can be found on a single leaf.

Larva (Figs 2, 3). The caterpillars are typical of those of Larentiinae. The head is rounded with a standard arrangement of stemmata, a spinneret that is long and pointed but not extending beyond the labium, and a retinaculum on each mandible. The thoracic prolegs each have 6–8 setae. Abdominal segments 1–5 are approximately twice as long as those posterior to A5. There are no ridges, bumps, swollen segments, scoli, or filaments. Abdominal segments exhibit typical larentiine chaetotaxy with a few exceptions: A2–A7 lack the extra L seta found in temperate larentiines, and A1 has an arrangement of setae that includes two D setae, three L setae, two SV setae, and one V seta. All spiracles are round with a single seta immediately dorsal. The abdomen bears prolegs only on A6 and A10; each A6 proleg has 5 setae, and A10 prolegs have 6 setae each. Crochets are biordinal and arranged in two groups that surround a large pad in a hemi-ellipse. There are paired paraprocts on A10, and the anal shield is rounded. Larvae scrape the bottoms of leaves, leaving characteristic windows of upper epidermal tissue on the host leaf.

Pupa (Fig. 4). The pupae of *Eois* are similar to those of other geometrids; they are 45–55 mm in length and dark brown. They are attached to the undersides of leaves by the hooked spines of the cremaster.

Imago. Adults of the *E.olivacea* species complex all share extremely similar wing color and pattern, with a pale yellowish to pale gray-green ground color, usually with one to three faint, jagged or wavy, narrow, whitish fasciae (i.e., antemedial, medial, and postmedial lines) extending from the costa to the hind margin of the forewing and continuing across the hindwing. The postmedial fascia is usually well defined, whereas the submedian and median fasciae are often reduced or lacking altogether (especially in worn specimens). The outer margin of the wings (termen) bears an extremely narrow line of red-brown to maroon scales, and the fringe is bright yellow throughout, in contrast to the wing ground color and the terminal line. There usually is a small brown dot near the apex of the discal cell in both the forewing and hindwing, but the dot is occasionally weakly expressed or absent.

On the head, the chaetosemata are represented by small, rounded patches posterior to the bases of the antennae, connected by a narrow, continuous row of setae located in a naked region near the posterior margin of the head, typical of members of the tribe Asthenini (Viidalepp 2011). The male genitalia are characterized by the absence of an uncus, with the semi-sclerotized scaphium occupying this position; the dorsal part of the tegumen narrow; the presence of lacina; and a patch of spines in the membrane surrounding the phallus, often arranged in two longitudinal rows. The vesica in all species examined bears a small, variable, semicircular plate with a saw-toothed margin (or field) around the curved side. Clusters of large, elongate cornuti are present in the vesica of a few species but absent in most. All these morphological features appear to be shared with members of the *E.goodmanii* and *E.muscosa* species complexes.

Members of the *E.goodmanii* species complex are superficially similar to those of the *olivacea* complex, but the wing ground color is usually a darker green or darker gray-green; the postmedial line is ill-defined and either dark rather than pale, extremely faint, or absent altogether; and the fringe is interrupted by brown patches rather than entirely yellow. Members of the *muscosa* species complex are superficially dissimilar to the other two groups, with the pale green forewing lacking medial and antemedial lines: instead with the inner two-thirds of the wing featuring a large, ill-defined patch of pale brown scales, and the fringe not contrasting with the forewing ground color.

**Description. Male. *Head***: Scales of frons smooth appressed, fawn brown; scales of vertex slightly paler; vertex between bases of antennae with narrow, transverse band of snow-white scales, separating fawn brown scales of frons from paler scales of vertex. Ocellus absent. Chaetosema a rounded patch posterior to base of antenna, with narrow continuous row across vertex in naked region near posterior margin of head. Compound eye large, comprising greater than 0.66 of head. Antenna cylindrical, bipectinate in males, with long, slender rami biciliate to tip, rami absent in distal 0.25 of antenna; dorsum of flagellomeres with white scales. Labial palpus with segment 2 approximately 0.5 length of segment 1; segment 3 short, approximately 0.25 as long as segment 1; length of all segments combined 0.5–0.7 × diameter of compound eye.

***Thorax***: Concolorous with forewing dorsum ground color. Legs long, slender, densely covered in scales, usually concolorous with thorax; tibia of mid- and hindlegs with conspicuous tibial spurs, midtibia with one, hind tibia with two, approximately 0.25 length of tarsomere 1; sclerotized tips of tibial spurs simple, elongated. Forewing broadly triangular, length 1.2 × width at termen, outer margin evenly convex, with discal cell less than 0.5 wing length, accessory cell long, originating from distal costal margin of discal cell. Ground color variable from pale yellow to pale greenish gray; antemedial line usually faint, wavy, ill defined, ivory; medial line either extremely faint or lacking altogether; postmedial line usually well defined, wavy, ivory, angled perpendicularly toward costa beyond M_3_; region from postmedial line to termen sometimes with a faint, narrow, zigzag, ivory line; discal spot usually well defined, somewhat oblong-round, red-brown; costal region usually faintly tinged pale pinkish brown, irregularly marked with small cream blotches; termen with narrow, dark red-brown line, concolorous with discal spot, variable from nearly straight to conspicuously scalloped. Fringe pale yellow. Forewing underside usually ivory, suffused with faint reddish brown, darkest in costal portion, with or without trace of dorsal pattern; discal spot round, orange-brown, faint to absent. Fringe pale yellow. Hindwing concolorous with forewing; antemedial line ill defined, ivory; postmedial line well defined, wavy, ivory; termen and fringe as in forewing. Hindwing underside ivory to yellowish gray, with ill-defined pattern similar to upperside; discal spot faint. Hindwing rounded, outer margin evenly convex, with discal cell approximately 0.33 as long as wing, M_3_ and CuA_1_ stalked; frenulum with one thick spine in male, 6–8 weaker spines in female.

***Abdomen***: Concolorous with thorax, usually with narrow row of white to cream scales at posterior end of each segment. Slender, extending beyond anal angle of hindwing. Tg8 somewhat narrower posteriorly; St8 slightly tapered posteriorly; St8 equal in width to St7; Tg8 roughly equal in width to Tg7; St8 equal in width to Tg7; posterior margin of St8 with shallow, U-shaped mesal excavation. Genitalia with uncus absent; tegumen narrow, dorsal part band-like, with arms forming rounded dorsal arch; lacina of variable size and shape from lateral margins of tegumen or transtilla; junction of tegumen and vinculum forming a shallow angle. Anal tube (scaphium) long, with ventral surface bearing a long, relatively wide, sclerotized band. Saccus shallow with transverse dorsal margin at base of valva; ventral margin forming small, transverse-ovoid pocket. Transtilla weakly sclerotized, distinctly bilobed. Membrane surrounding phallus with one or two variable fields of short spines. Juxta gradually narrowing dorsally with wide dorsal margin and U-shaped mesal excavation. Area between phallus and juxta simple. Valva elongate-subrectangular to rounded, with dorsal and ventral margins roughly parallel, but ventral margin variably constricted near middle, with patch of long, fine setae at constriction; costa long, narrow, band-like, extending to apex. Apex of valva with fine, hair-like setae, similar to those covering remainder of valva; sacculus narrow, lightly sclerotized, 0.3–0.5 as long as valva, ventral margin contiguous with ventral margin of valva, without secondary group of robust setae near apex of sacculus; inner margin of sacculus lacking row of setae. Phallus usually about as long as valva, wide, with broad distal opening, narrowed basally, with rounded base; apex developed into blade-like ventral process. Vesica with at least one semicircular plate with a saw-toothed margin; cornuti variable: two clusters of fine short cornuti, a single group of large, spine-like cornuti, or cornuti absent altogether; base of vesica minutely scobinate with a pair of narrow, curved scobinate sclerites.

**Female. *Head***: Essentially as described for male, but antenna filiform, lacking rami.

***Thorax***: Essentially as described for male.

***Abdomen***: Essentially as described for male. Genitalia with papillae anales narrow to roughly triangular, distal portion rounded. Tg8 either narrow, quadrate, or U-shape, with transverse posterior margin, bearing transverse striations. Dorsal membrane between Tg8 and papillae anales simple, with a small membranous invagination. Posterior apophyses longer than anterior apophyses. Ostium forming a large, dorso-ventrally compressed, vase-like structure. Region between ostium and ductus bursae broadly membranous, bearing a ventral appendix. Ductus bursae narrow, lightly sclerotized with lateral margins rolled upward, ductus U-shaped in cross-section. Ductus bursae arising from a small, narrow appendix at base of corpus bursae ventrally, curving to right. Corpus bursae oblong with variable lateral band of long spines; rounded anteriorly, membranous, continuous with remainder of corpus. Signum horn-like with base partially protruding beyond outer wall on left side of corpus bursae. Internal part of signum comprised of long, curved spines. Narrow, strap-like, spinose sclerite arising from signum, wrapping around corpus bursae; remainder of corpus bursae beyond signum smooth and simple. Corpus bursae often composed of two parts, with smooth distal portion broadly attached to remainder of corpus.

Few members of the species complex can be distinguished reliably based on facies alone. However, structures of the male and female genitalia provide morphological characters for separating these similar-looking adults. The species complex can be divided into two subgroups based on the arrangement and size of the cornuti in the vesica of the male phallus: Group I species have either one or two small patches of small cornuti (usually less than 0.15 the length of the phallus) or lack them altogether; and Group II species have one or two patches of large, elongate, robust cornuti that are >0.25 the length of the phallus (Doan 2023). We treat only Group I species here.

#### ﻿ Eois olivacea

Taxon classificationAnimaliaLepidopteraGeometridae

(Felder & Rogenhofer, 1875)

1EF12110-61B4-5034-B675-B5C87FB9143A

Figs 5–7

Jodis olivacea Felder & Rogenhofer, 1875: pl. 128, fig. 13.
Eois olivacea : Parsons et al. 1999: 279; Brehm et al. 2011: 1106.

##### Type material examined.

***Holotype*** ♂, Colombia, Bogota [ca 2630 m] (NHMUK).

##### Additional specimens examined.

Colombia: Fasaogasuga, [1770 m], [no date] (1♂), Dognin Collection, USNM slide 154,479, USNM ENT 01906870 (USNM).

##### Remarks and diagnosis.

**Figures 5–7.**
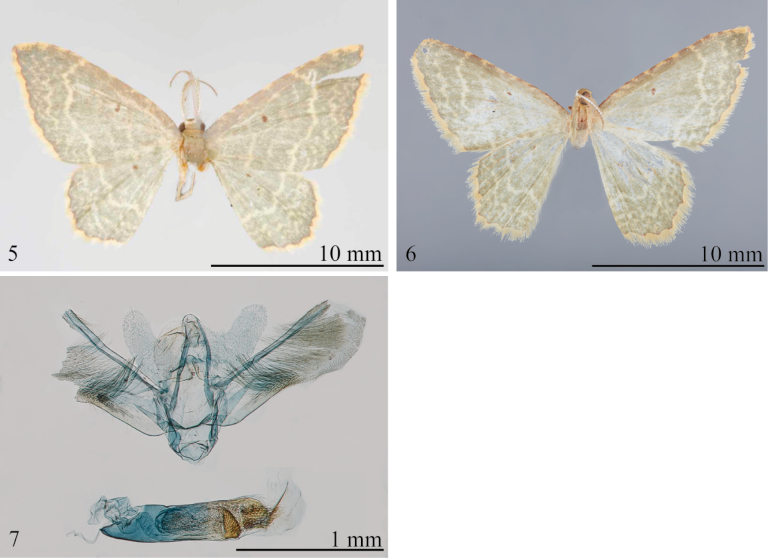
*Eoisolivacea* adult and male genitalia **5** holotype upperside (NHMUK) **6**E.cf.olivacea from Colombia upperside (USNM) **7** male genitalia of “surrogate” *E.olivacea*, USNM slide 154,479.

The holotype male of this species (Fig. 1) lacks an abdomen; hence, comparisons of the genitalia to congeners is impossible. However, among several candidates from Colombia (USNM) that are potential conspecifics of *E.olivacea*, a male from Fasaogasuga possesses the distinctive jagged line midway between the postmedial line and the termen of the forewing that is characteristic of the type of *E.olivacea* (Fig. 5). Hence, we provisionally assign that specimen to *E.olivacea*. The question may be resolved through molecular analyses that are beyond the scope of this contribution. In the redescription below, details of external features are based on the holotype, and those of the genitalia are based on the putative conspecific. Although the genitalia of the specimen from Fasaogasuga are damaged, the important characters are intact.

If our association is correct, *E.olivacea* has the simplest vesica of any member of the complex, with the possible exception of *E.beebei*, with a single semicircular plate bearing teeth along the curved margin. The lacina of *E.olivacea* are shorter than those of *E.pseudolivacea*.

##### Redescription.

**Male. *Head***: Essentially as described for species complex. ***Thorax***: Essentially as described for species complex, except forewing length 9.0 mm (*n* = 1); forewing ground color pale gray-green; antemedial and medial lines faint, postmedial line well defined, ivory; distinctive, ivory, zigzag line midway between postmedial line and termen; discal spot small; costal region lightly tinged pale pinkish brown, irregularly and faintly marked with small cream blotches; termen with narrow, dark red-brown line, concolorous with discal spot, consisting of uninterrupted series of inward-directed scallops. Forewing underside as described for species complex. Hindwing concolorous with forewing, with antemedial line ill defined, postmedial line well defined; termen and fringe as in forewing. ***Abdomen***: Genitalia (Fig. 7) with tegumen slender, lacina broad basally, upcurved, narrower and somewhat parallel-sided in distal 0.5, rounded apically; valva subrectangular, slightly constricted near middle with patch of long fine setae at constriction, sacculus well defined, terminating at constriction of valva, outer margin weakly angled subbasally; phallusca as long as valva; vesica with semicircular, saw-toothed plate, lacking large cornuti; membrane surrounding phallus with a pair of dorsal fields of short spines.

**Female.** Unknown.

##### Distribution and biology.

This species is known from the holotype from near Bogotá and a second specimen from Fasaogasuga, Colombia. The early stages remain unknown.

#### ﻿ Eois pseudolivacea

Taxon classificationAnimaliaLepidopteraGeometridae

Doan sp. nov.

7EEC2C3E-55BD-5507-A58E-77CDBAB48818

https://zoobank.org/E3FD5A8C-2186-4512-957A-1B9BCFAF790E

Figs 8–11

##### Type material.

***Holotype*** ♂, Ecuador, Napo, Yanayacu Biological Station, 2163 m, 00°35'0.9"S, 077°53'0.4"W, Mar 2010, r.f. 46067, Earthwatch, slide 69575 (UNR). ***Paratypes*** (5♂, 6♀). Ecuador: Napo: Yanayacu Biological Station, 2066.8 m, 00°34'0.001"S, 77°52'.001"W, Jun 2013, r.f. 75714, 75718 (2♂), July 2013, r.f. 78585, 78550, 78563 (1♂, 2♀), Sept 2014, r.f. 86500 (1♀), Aug 2015, r.f. 88081 (1♂), Earthwatch (UNR). Ecuador: Napo: Yanayacu Biological Station, 2096.6 m, 00°35'7.02"S, 77°52'31.379"W, Oct 2013, r.f. 80578, 80648, 80696 (3♀), Earthwatch (UNR). [no further locality data] r.f. B1409 (1♂), Earthwatch (UNR).

##### Remarks and diagnosis.

**Figures 8–11.**
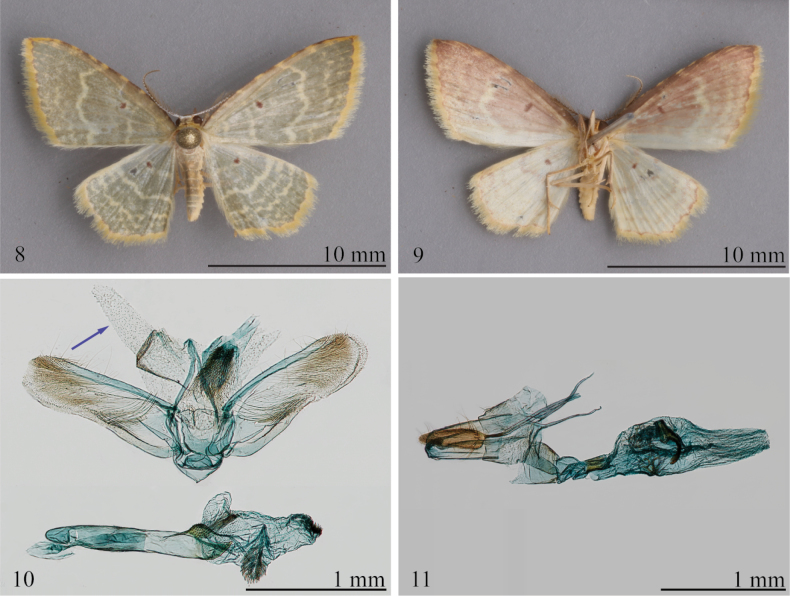
*Eoispseudolivacea* adult and genitalia **8** holotype upperside (UNR) **9** holotype underside (UNR) **10** male genitalia, slide 69575 (UNR); arrow indicates elongate lacina **11** female genitalia, slide 86478 (UNR).

*Eoispseudolivacea* is described from Ecuador, where it occurs in sympatry with several very similar congeners. Superficially, *E.pseudolivacea* is nearly indistinguishable from other members of the species complex (Figs 8, 9). However, the male genitalia (Fig. 10) are easily recognized by the length of the lacina, which is nearly as long as the valva.

##### Description.

**Male. *Head***: Essentially as described for species complex. ***Thorax***: Essentially as described for the species complex, except forewing length 9.5 mm (*n* = 12); forewing ground color pale moss; antemedial line faint, ivory, uniform in width throughout; medial line faint, wavy, ivory; postmedial line prominent, well defined, wavy, ivory, perpendicularly angled toward costa beyond M_3_; region from postmedial line to termen with two narrow, wavy, ivory lines; discal spot well defined, round, red-brown; costal margin banded cream and dark red-brown; termen with narrow, dark red-brown line, concolorous with discal spot. Fringe pale yellow. Forewing underside ivory, suffused with faint reddish brown, with faint trace of dorsal pattern; discal spot round, orange-brown, faint to absent. Fringe pale yellow. Hindwing concolorous with forewing; antemedial line ivory; postmedial line wavy, cream, perpendicularly angled toward costa beyond CuA. Hindwing underside with pattern similar to upperside, with faint red-brown antemedial and postmedial lines, region from postmedial line to termen with two, wavy, red-brown lines; discal spot faint. ***Abdomen***: Genitalia (Fig. 10) with lacina nearly as long as valva, somewhat parallel-sided; membrane surrounding phallobase bearing large dorsal field of short spines arranged in two longitudinal rows; vesica bilobed, each lobe with distal group of small spine-like cornuti.

**Female. *Head*** and ***Thorax***: Essentially as described for male, but antenna slightly narrower, lacking rami. ***Abdomen***: Genitalia (Fig. 11) with papillae anales narrow; ductus bursae narrow; corpus bursae oblong, with large, curved, spinelike signum located laterally on left side.

##### Distribution and biology.

This species is known only from Napo Province, Ecuador. It was reared from larvae discovered on *Piperlanceifolium* (*n* = 456) and *P.baezanum* (*n* = 49).

The eggs, larvae, and pupae of *E.pseudolivacea* have all the general characteristics described above for *Eois* with no modifications (Figs 1–4). First and second instars are similarly colored, with a clear beige head capsule, yellowish green thorax, abdomen, pinacula, and setae. The thoracic legs and prolegs are clear. Instars 3–5 have this same color pattern but also have paired broad cream patches subdorsally, extending across all segments from T2 to A8; A9 and A10 are usually pale cream colored, pinacula are chalky white with brown setae, and mandibles are dark brown. A thin white lateral stripe connects all spiracles. Pupae are usually pressed along major leaf veins on the underside of the leaf.

##### Etymology.

The species name refers to the superficial similarity of this species to *E.olivacea*.

#### ﻿ Eois auruda

Taxon classificationAnimaliaLepidopteraGeometridae

(Dognin, 1900)

660D04C2-92E3-5261-AFF7-4288FD40C48D

Figs 12–14

Amaurinia auruda Dognin, 1900: 443.
Eois auruda : Parsons et al. 1999: 279; Brehm et al. 2011: 1106.

##### Type material examined.

***Holotype*** ♂, Ecuador, [Loja Province], environs de Loja, 1889, Dognin Collection, USNM type 32227, USNM ENT 01906872 (USMN). ***Paratype*** (1♂). Ecuador, Loja, valley of Zamora [00°35.9'S, 77°53'W, 2163 m], May 1886, USNM slide 154,179, USNM ENT 01906873 (USMN).

##### Additional material examined.

Ecuador: [Loja Province], Environs de Loja, 1891 (1♂), USNM slide 154,650, USNM ENT 01906874 (USNM).

##### Remarks and diagnosis.

**Figures 12–14.**
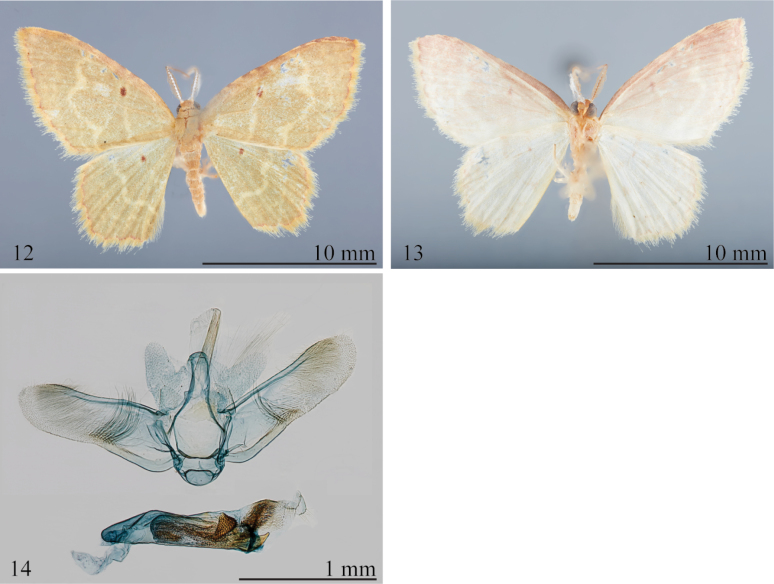
*Eoisauruda***12** holotype upperside (USNM) **13** holotype underside (USNM) **14** male genitalia, USNM slide 154,179.

*Eoisauruda* was described by Dognin based on two males from “Loja et vallée de Zamora, Equateur.” One specimen is clearly labelled “type” and the other “co-type.” Superficially, *E.auruda* is paler yellowish orange than the pale green of most members of the species complex, and the maroon scallops of the termen are weakly interrupted by yellowish brown (Fig. 12). In the male genitalia (Fig. 14), the membrane surrounding the phallus has a smaller dorsal field of short spines than in related species.

##### Description.

**Male. *Head***: Essentially as described for complex. ***Thorax***: Essentially as described for complex, except forewing length 9.0–10.0 mm (*n* = 3). Forewing ground color pale yellowish green; postmedial line prominent throughout; antemedial line faint; discal spot, small, round, faint, reddish brown; costal region slightly tinged with pale brown; termen with dark maroon scalloped line, concolorous with discal spot; fringe two-toned, mostly pale yellow with small incursions of red and/or brown between veins M_2_ and M_3_. Forewing underside pale yellow, suffused with pale reddish brown, with faint indication of upper surface markings; antemedial line absent. Discal spot round, faint to absent, orange-brown; termen with dark orange-brown line. Hindwing ground color brownish pale yellowish green; antemedial line absent or very faint; postmedial line well defined; region between postmedial line and termen with several extremely faint, interrupted wavy, pale-yellow lines; discal spot well defined, red-brown. Fringe two-toned, mostly yellow, interrupted by pale red veins M_2_ and M_3_. Hindwing underside with pattern and coloration similar to forewing underside, but with pale pinkish tint, especially in costal region. ***Abdomen***: Essentially as described for genus. Genitalia (Fig. 14) with tegumen slender, lacina broad basally, upcurved, somewhat parallel-sided in distal 0.5; valva subrectangular, slightly constricted near middle with patch of long fine setae, sacculus well defined, rounded subbasally, terminating at constriction of valva; phallus long, broad; vesica with semicircular, saw-toothed plate, lacking large cornuti; membrane surrounding phallus with very small dorsal field of short spines.

**Female.** Unknown.

##### Distribution and biology.

This species is known from three specimens from Loja Province, Ecuador, collected before the turn of the twentieth century.

#### ﻿ Eois beebei

Taxon classificationAnimaliaLepidopteraGeometridae

Fletcher, 1952 stat. rev.

E71494D9-18B2-5E2C-9422-686FD1FDF33B

Figs 15 , 16

Racheospila beebei Fletcher, 1952: 101.
Eois beebei : Parsons et al. 1999: 279; Brehm et al. 2011: 1106.

##### Type material examined.

***Holotype*** ♂, Venezuela, Rancho Grande near Maracay, W. Beebe, No. 481604 (NHMUK).

**Figures 15, 16.**
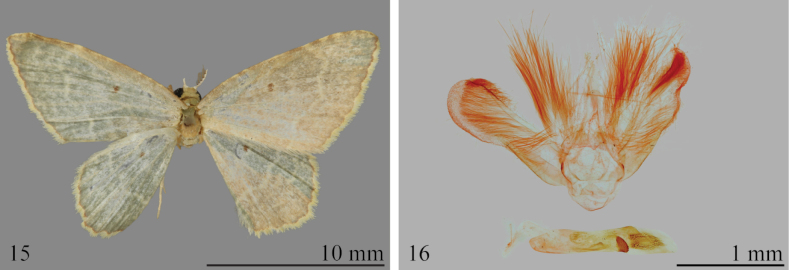
*Eoisbeebei***15** holotype upperside (NHMUK) **16** male genitalia (NHMUK).

##### Remarks and diagnosis.

Fletcher (1952) described this species from a single male collected by William Beebe at Rancho Grande (now known as Henri Pittier National Park), in the Venezuelan Costal Range, an historically well-known collecting locality. Fletcher’s description is somewhat outdated, as is his rather crude drawing of the male genitalia. The species was treated as a synonym of *E.olivacea* by Parsons et al. (1999), without the benefit of a comparison of the genitalia with those of the latter.

As in many members of Group I, the phallus of *E.beebei* has a large, conspicuous, scobinate plate with a saw-toothed edge situated near the distal end of the vesica, but lacks long cornuti (Fig. 16). The species can be distinguished from *E.olivacea* by the shorter and narrower valvae, and from *E.pseudolivacea* by the shorter lacina.

##### Redescription.

**Male. *Head***: Frons and vertex pale pinkish buff with distinct white bar between bases of antennae; labial palpus pale pinkish buff, length~ 0.5 diameter of compound eye; pectinations of antenna ~ 4 × as long as the diameter of the shaft. ***Thorax***: Pale olive; forewing ground color pale olive, anterior 0.5 irrorate with pale grayish brown, costa lightly irrorated with cream-brown, postmedial fascia white, discal spot fuscous. Fringe chalcedony yellow. Forewing undersurface white, glossy; discal spot minute. ***Abdomen***: Pale olive, each segment edged posteriorly with white. Male genitalia (Fig. 16) with top of tegumen broadly rounded; lacina supporting long androconial scales; valva subrectangular with distinct sacculus along venter of basal 0.5. Phallus with weakly sclerotized patch near apex; vesica with two scobinate plates in apical 0.5, lacking elongate cornuti.

**Female.** Unknown.

##### Distribution and biology.

Known only from the type locality.

#### ﻿ Eois boliviensis

Taxon classificationAnimaliaLepidopteraGeometridae

(Dognin, 1900)

A9F3D2E1-9568-50BD-9DA8-85D8CCA3225D

Figs 17–19

Thalassodes boliviensis Dognin, 1900: 215.
Eois boliviensis : Parsons et al. 1999: 275; Brehm et al. 2011: 1105.

##### Type material examined.

***Holotype*** ♂, Bolivia, [no additional data], USNM slide 154,454, USNM ENT 01906882 (USNM).

##### Remarks and diagnosis.

Dognin (1900) described this species from a single male from Bolivia, without a specific locality. There are two additional specimens in the USNM from the Dognin collection identified by him as *boliviensis*. This species was transferred to *Amaurinia*, now considered a synonym of *Eois*, and later treated as *Eois* by Parsons et al. (1999) and Brehm et al. (2011).

**Figures 17–19.**
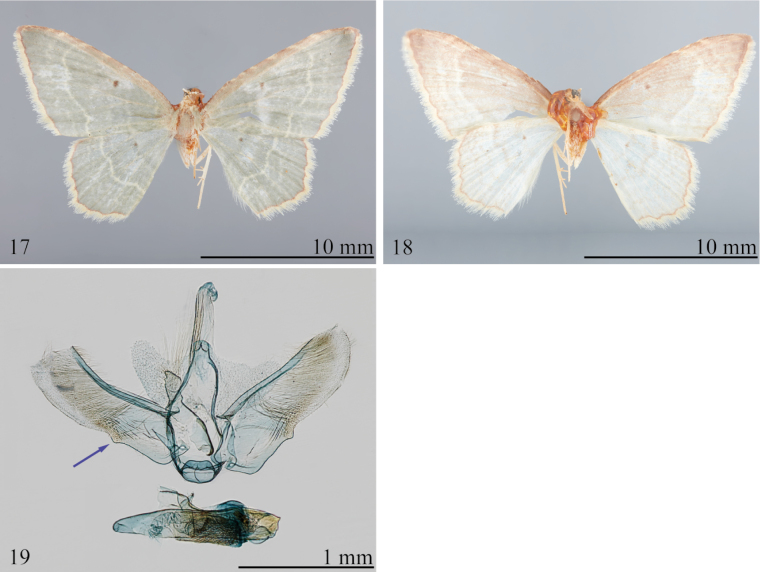
*Eoisboliviensis***17** holotype upperside (USNM) **18** holotype underside (USNM) **19** male genitalia, USNM slide 154,454; arrow indicates sacculus with small triangular process at termination.

*Eoisboliviensis* is superficially similar to other members of the species group, but the ground color is a distinctive darker gray-green (Fig. 17). The male genitalia (Fig. 19) are also typical of other species, with a coarsely toothed, scobinate plate near the distal end of the vesica, and the absence of long cornuti. The species can be distinguished from all other members of the species complex by the small, free, triangular lobe at the distal end of the sacculus~ 0.33 the distance from the base to the apex of the valva (Fig. 19), the relatively smaller toothed plate in the vesica, and the reduced patch of spines in the membranous region surrounding the phallus in the male genitalia.

##### Redescription.

**Male. *Head***: Frons and vertex pale green, with white bar between bases of antennae; labial palpus pale grayish green. ***Thorax***: Essentially as described for species complex, except forewing length 10.0 mm (*n* = 1); forewing ground color gray-green, with straw colored, wavy, postmedial line; small spot in cell, red-brown; terminal line more wavy than scalloped, reddish brown; fringe straw. Underside pale gray-green, entirely suffused with pale reddish brown. Hindwing ground color and postmedial line concolorous with those of forewing. Underside pale grayish green, with pinkish tint in costal region. ***Abdomen***: Pale green. Male genitalia (Fig. 19) with tegumen arms joined dorsally forming a weakly bilobed process; lacina broad in basal 0.4, narrower and weakly attenuate in distal 0.6 with rounded outer margin, bearing long, hair-like androconial scales; valva elongate-subrectangular, ~ 3 × longer than wide, parallel-sided, with rounded outer margin; sacculus angled subbasally, with small triangular process at termination,~ 0.33 distance from base to apex of valva; phallus ca as long as valva, attenuate basally, somewhat truncate apically; membrane surrounding phallus with broad, weakly developed field of spines; vesica with small, coarsely toothed plate in apical 0.5, lacking elongate cornuti.

**Female.** Unknown.

##### Distribution and biology.

*Eoisboliviensis* is known from three specimens collected in Bolivia without additional locality data. Nothing is known of the biology.

#### ﻿ Eois parumsimii

Taxon classificationAnimaliaLepidopteraGeometridae

Doan sp. nov.

AC888F6E-C3F6-588F-9EEA-B351452E904E

https://zoobank.org/4A65A58B-A430-4E22-A1F0-79D75A7267F8

Figs 20–23

##### Type material.

***Holotype*** ♂, Ecuador, Napo, Yanayacu Biological Station, 2113.9 m, 00°35'48.998"S, 77°53'17.998"W, Nov 2012, r.f. 71361, Earthwatch (UNR). ***Paratypes*** (5♂, 2♀). Ecuador: Napo: Yanayacu Biological Station, 2066.8 m, 00°34'0.001"S, 77°52'0.001"W, Feb 2005, r.f. 1673, 1674 (2♂), Sept 2010, r.f. 51792 (1♂), Earthwatch (UNR); 2188.4 m, 00°35'54"S, 77°53'44.34"W, Nov 2005, r.f. 9442 (1♂), Earthwatch (UNR); 1240.7 m, 00°43'38.798"S, 77°46'22"W, Jun 2014, r.f. 84256 (1♀), Earthwatch (UNR); 1871.9 m, 00°31'31.2"S, 77°52'35.399"W, Aug 2014, r.f. 85960, 85963 (1♂, 1♀), Earthwatch (UNR).

##### Diagnosis.

**Figures 20–23.**
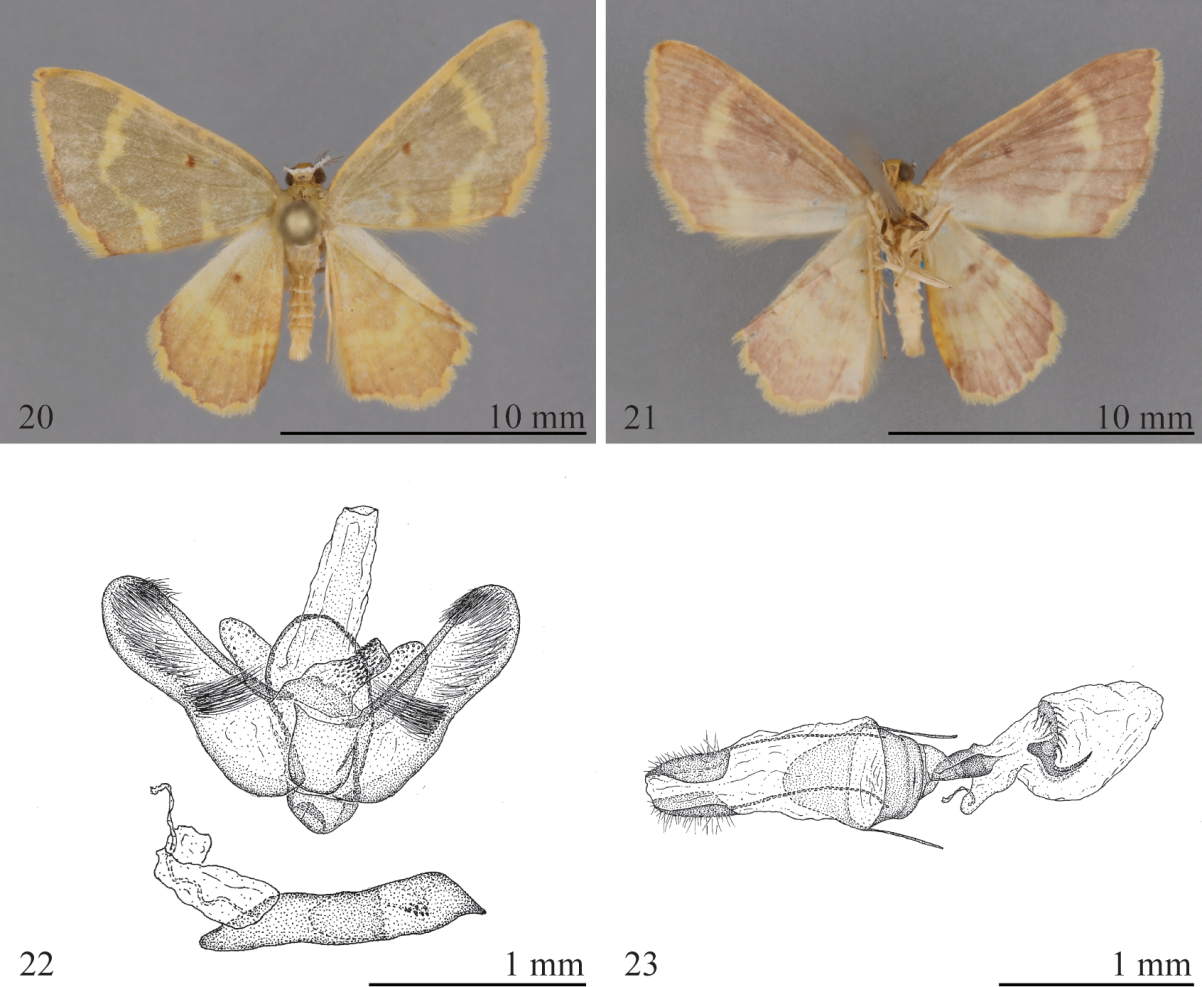
*Eoisparumsimii***20** holotype upperside (UNR) **21** holotype underside (UNR) **22** male genitalia, slide 75819 (UNR) **23** female genitalia, slide 75961 (UNR).

This species is described from specimens reared from larvae collected at Yanayacu Biological Station in Ecuador. Externally, *E.parumsimii* is distinct from all of species in the genus, with a much broader, yellow, postmedial line on a pale pinkish gray ground color (Fig. 20). The male genitalia of *E.parumsimii* can be distinguished from those of other members of the complex by the following combination of character states: the ventral margin of the saccus forming a blunt conical pocket (vs a small, transverse-ovoid pocket in most other species); and the membrane surrounding the phallus with a large dorsal field of short spines arranged in a series of longitudinal rows (vs arranged in two longitudinal rows most other in species). The female genitalia of *E.parumsimii* have short, trapezoidal papillae anales, whereas the papillae anales are slenderer in most other species; and the signum is located laterally on the left side of the corpus bursae, whereas it located ventrally in many other species of the complex.

##### Description.

**Male. *Head***: Essentially as described for species complex. ***Thorax***: Essentially as described for species complex, except forewing length 8.0–9.0 mm (*n* = 8); ground color manzanilla olive, anterior portion of antemedial line very faint, posterior portion prominent, pale yellow; postmedial line well defined, wavy, pale yellow; discal spot well defined, red brown; basal 0.66 of costal margin concolorous with ground color, distal portion with clay-brown markings; termen with slender clay-brown line; fringe two-toned, mostly pale yellow, with red-brown incursion between veins M_2_ and M_3_. Forewing underside ground color pale yellow, suffused with red-brown, inverse to dorsal pattern; antemedial line absent; postmedial line prominent, width variable, but broader near costa and posterior margin; discal spot round, faint, clay-brown; termen clay brown. Fringe pale yellow. Hindwing upperside ground color clay-brown; antemedial line faint; postmedial line wavy, pale yellow; discal spot round, well defined, orange-brown; termen clay-brown. Fringe pale yellow. Hindwing with pattern similar to forewing, with prominent pale yellow antemedial and postmedial lines and faint discal spot. ***Abdomen***: Genitalia (Fig. 22) with tegumen arms forming rounded triangular dorsal arch, curving slightly posterad; ventral margin of saccus forming a blunt conical pocket; transtilla slender, V-shaped; juxta wide basally, abruptly narrowed in distal 0.33 with acute dorsal part; dorsal margin of juxta narrow, truncate, with a small down-curved lip; valva subrectangular, weakly constricted on ventral margin near distal end of sacculus; a brush of bristle-like setae near apex, contrasting with remaining setae; setae at apex of sacculus longer than width of valva; ventral margin of sacculus bowed outward; base of phallus narrow, horn-shaped, membrane surrounding phallus with large dorsal field of short spines arranged in longitudinal rows; phallus ca as long as valva; vesica bifurcated distally with two appendices, one with distal group of spine-like cornuti, the other at base of vesica with a single, large scobinate plate.

**Female. *Head*** and ***Thorax***: Essentially as described for male except lacking rami on antenna. **Abdomen**: Genitalia (Fig. 23) with papillae anales short, trapezoidal. Dorsal membrane between Tg8 and papillae anales bearing a large dorsal sac. Ductus bursae wide; ductus seminalis arising from elongate, triangular appendix at base of corpus bursae; Corpus bursae oblong, without mesal constriction, distal appendix absent; signum wing-shaped with serrate lateral margins, located laterally on left side of corpus bursae, internal part horn-like.

##### Biology and distribution.

This species is known only from Napo Province, Ecuador. Adults were reared from larvae (*n* = 25) discovered in the field on *Piperbaezanum*, which is a threatened species endemic to Ecuador (Santiana and Pitman 2004).

The eggs, larvae, and pupae of *E.parumsimii* have all the same general characteristics described above for *Eois* with no modifications (Figs 1–4) and are difficult to distinguish from those of *E.pseudolivacea*. All larval instars (until the prepupal stage) are similarly colored, with a translucent beige head capsule, pale green thorax and abdomen, translucent pinacula, and paired subdorsal yellow spots on all segments form T2–A6. Abdominal segments are separated by slight constrictions. The prothoracic legs and abdominal prolegs are tan. The prepupa is translucent.

##### Etymology.

The species epithet *parumsimii* is a patronym for Michael Lumibao, who is the partner and long-time supporter of the first author; the name is derived from his Chinese zodiac animal.

## Supplementary Material

XML Treatment for Eois olivacea

XML Treatment for Eois pseudolivacea

XML Treatment for Eois auruda

XML Treatment for Eois beebei

XML Treatment for Eois boliviensis

XML Treatment for Eois parumsimii

## ﻿Additional information

### Author ORCIDs

Lydia M. Doan https://orcid.org/0000-0002-7039-8814

John W. Brown https://orcid.org/0000-0001-5610-9855

Matthew L. Forister https://orcid.org/0000-0003-2765-4779

Lee A. Dyer https://orcid.org/0000-0002-0867-8874

### Data availability

All of the data that support the findings of this study are available in the main text.

## ﻿Appendix 1

**Adult morphological characters in *Eois***

This appendix contains additional methodology of *Eois* species identification, complete with a morphological guide based off of 94 *Eois* and 5 outgroup *Eois* species, as well as character matrix of species *E.parumsimii* and *E.pseudolivacea* (Table A1). The guide includes 107 characters (37 binary and 70 multi-state), with 16 external characters (characters 1–16), 58 male-only characters (characters 17–75), and 33 female-only characters (characters 76–107).

Methodology, terminology, and abbreviations

Examination of specimens was performed under a Zeiss Stemi 2000-C stereomicroscope with SCHOTT EasyLED ring-light illuminator. Species were identified through the examination of photos of holotype or type photos (Brehm unpublished data) and their genitalia. Terminology for genital structures and components of forewing pattern follow Holloway (1997) and Viidalepp (2011). With the exception of an unusual structure in the male genitalia of members of the several clades of *Eois*, that appears to lack a term. It is a flat, membranous, lateral flap of variable size and length attached to the sides of the tegumen and/or transtilla, which supports long, fine male scent scales or androconia. The term “lacina” is suggested for these structures.

In the guide below, brief descriptions of each morphological character are followed by a number/score, or character state, in brackets (e.g. [**1**], [**2**], [**3**], etc.) which denotes variation of that character. Scores of 0, unless otherwise stated, are character states commonly observed in the outgroup taxa.

Abbreviations for morphological structures in the guide are as follows: **Lp1** = labial palpus segment 1; **Lp2** = labial palpus segment 2; **Lp3** = labial palpus segment 3; **FW** = forewing; **HW** = hindwing; **Tg7** = tergum 7; **Tg8** = tergum 8; **St7** = sternum 7; **St8** = sternum 8; **PVP** = postvaginal plate; **DB** = ductus bursae; **DS** = ductus seminalis; **CB** = corpus bursae.

**External characters of both genders**

1. Scales between antennal bases concolorous with vertex of head [**0**]; a transverse band of snowy white scales located between antennal bases [**1**]; a narrow band of creamy yellow scales between antennal bases [**2**].
2. Dorsum of antennal shaft/flagellum concolorous with remainder of head [**0**]; dorsum of antennal shaft white [**1**]; each antennal annulation mostly brown, edged with lighter scales [**2**]; dorsum of antennal shaft with alternating rings of light brown and creamy yellow scales [**3**]; each antennal annulation a mixture of brown and cream-colored scales [**4**]; mesal surface of antennal shaft white, lateral surface brown [**5**].
3. Labial palpus segment 2 roughly half as long as Lp1 [**0**]; Lp2 roughly equal in length to, or slightly longer than, Lp1 [**1**]; Lp2 over twice as long as Lp1 [**2**].
4. Labial palpus segment 3 small, approximately ¼ as long as Lp1 [**0**]; Lp3 somewhat elongate, nearly ½ as long as Lp1 [**1**].
5. Forewing lacking shiny scales [**0**]; forewing with patches of glossy, metallic scales, especially grouped within dark-patterned areas [**1**].
6. Forewing lacking a spot near apex of discal cell [**0**]; FW with a small brown spot near apex of discal cell [**1**].
7. Hind wing lacking a spot near apex of discal cell [**0**]; HW with a brown spot near apex of discal cell [**1**].
8. Forewing discal cell at least ½ wing length [**0**]; FW discal cell much shorter than ½ wing length [**1**].
9. Hind wing discal cell at least ½ as long as wing, M3 and CuA1 veins arising separately from corner of cell [**0**]; HW discal cell short, approximately 1/3 as long as wing, M3 and CuA1 stalked [**1**].
10. Forewing accessory cell present, moderate in length [**0**]; accessory cell present, long [**1**]; FW accessory cell absent [**2**].
11. Forewing outer margin evenly convex [**0**]; FW outer margin with a projection at apex of vein CuA1 [**1**].
12. Hindwing outer margin evenly convex [**0**]; HW outer margin with a projection at apex of vein M3 [**1**].
13. Male forewing shorter than female FW [**0**]; male forewing longer than female FW [**1].**
14. Sclerotized tips of tibial spurs short and delicate, simple [**0**]; sclerotized tips of tibial spurs elongate, simple [**1**]; sclerotized tips of tibial spurs extremely long, with blade-like lateral margins [**2**]; sclerotized tips of tibial spurs minute [**3**].
15. Ventral margin of tibial spur simple [**0**]; each tibial spur with a sclerotized, scaleless seam running along ventral surface [**1**].
16. Dorsum of abdomen a single, uniform color [**0**]; abdominal dorsum yellow with a reddish stripe on the posterior margin of each segment [**1**]; abdominal dorsum yellow with a wide transverse stripe on segments 3+4 and a second stripe on 7+8 [**2**]; abdominal dorsum with a single spot on anterior margin of each segment [**3**]; abdominal dorsum pink with a yellow mesal spot on segment 2 and one on segment 3 [**4**]; abdomen yellowish, with a brown dorsal spot on segment 2 [**5**].

Male-Only Characters

1. Each antennal annulation, exclusive of rami, roughly cylindrical [**0**]; antenna fasciculate [**1**].
2. Antenna bipectinate nearly to apex, rami becoming gradually shorter distally [**0**]; antenna bipectinate in basal ¾, simple in distal ¼ [**1**]; antenna bipectinate in basal 2/3, simple in distal 1/3 [**2**]; rami absent, antenna ciliate [**3**].
3. Rami of antenna relatively short [**0**]; rami of antenna long and thin [**1**]; rami extremely short and flattened, apices bearing long bristles [**2**]; rami absent [?].
4. Tergum 8 roughly rectangular [**0**]; Tg8 somewhat narrower posteriorly [**1**]; tergum 8 strongly tapered posteriorly [**2**].
5. Tergum 8 roughly equal in width to Tg7 [**0**]; Tg8 much narrower than Tg7 [**1**].
6. Posterior margin of tergum 8 simple [**0**]; posterior margin of tergum 8 bearing a row of golden, bristle-like scales [**1**]; posterior margin of Tg8 with a small, somewhat sclerotized upturned flange [**2**].
7. Sternum 8 roughly rectangular [**0**]; sternum 8 slightly tapered posteriorly [**1**]; sternum 8 strongly tapered posteriorly [**2**].
8. Sternum 8 equal in width to St7 [**0**]; Sternum 8 much narrower than St7 [**1**].
9. Posterior margin of St8 transverse [**0**]; posterior margin of St8 with a shallow, U-shaped mesal excavation [**1**]; posterior margin of St8 with a deep, V-shaped mesal excavation [**2**]; St8 asymmetrical, divided into two asymmetrical parts [**3**].
10. Posterolateral angles of St8 simple [**0**]; posterolateral angles of St8 acute [**1**]; posterolateral angles of St8 forming elongate prongs [**2**].
11. Uncus present [**0**]; uncus absent [**1].**
12. Lateral portion of tegumen arms narrow [**0**]; lateral portion of tegumen arms extremely wide [**1**].
13. Junction of tegumen and vinculum forming a shallow angle [**0**]; junction of tegumen and vinculum forming a deep notch [**1**].
14. Dorsal part of tegumen, where arms meet at midline, relatively wide [**0**]; dorsal part of tegumen, where arms meet at midline, narrow, band-like [**1**]; dorsal part of tegumen extremely narrow [**2**].
15. Dorsal portion of tegumen, where arms meet at midline, forming a rounded arch [**0**]; tegumen arms forming a somewhat triangular dorsal arch, curving slightly backward [**1**]; tegumen arms forming a narrow dorsal arch, curving strongly backward [**2**].
16. Arms of tegumen fully sclerotized, androconia absent [**0**]; each arm of tegumen with a membranous section along posterior margin, this bearing a set of long androconia [**1**]; androconia on arms of tegumen attached to a short lacina (< ½ as long as valva) [**2**]; androconia of tegumen attached to a long lacina, nearly ½ as long as valva [**3**]; lacina at least 2/3 as long as valva [**4**].
17. Anal tube short, with a short longitudinal ventral sclerite [**0**]; anal tube long, ventral surface bearing a long, relatively wide sclerotized band [**1**]; anal tube extremely long, extending nearly to apex of valvae, ventral surface bearing a narrow longitudinal band [**2**].
18. Surface of sclerite below anal tube simple [**0**]; sclerite below anal tube with a longitudinal furrow [**1**].
19. Region of membrane below saccus simple [**0**]; region of membrane below saccus with a small pocket, bearing a set of spatulate androconia [**1**].
20. Saccus deep, dorsal margin partially enclosing bases of valvae [**0**]; saccus shallow, dorsal margin transverse, valva bases exposed [**1**].
21. Ventral margin of saccus broadly triangular [**0**]; ventral margin of saccus gently curved, band-like [**1**]; ventral margin of saccus forming a small, transverse-ovoid pocket [**2**]; ventral margin of saccus forming a blunt conical pocket [**3**]; ventral margin of saccus forming an acute, triangular pocket [**4**]; ventral margin of saccus quadrate [**5**].
22. Transtillar arms meet at midline to form a small U-shaped structure, pointing anteriorly [**0**]; transtillar arms forming a large, shelf-like, U-shaped structure anteriorly [**1**]; transtillar arms forming a small, V-shaped structure [**2**].
23. Transtillar arms lacking projections (most *Eois*) [**0**]; each transtillar arm bearing an elongate, setose process (“labides”) (outgroup character) [**1**]; transtilla absent [?].
24. Area of manica posterior to tegumen simple [**0**]; area of manica posterior to tegumen bearing a pocket of long, bristle-like scales [**1**].
25. Sclerite connecting base of valval costa to dorsum of juxta relatively narrow, simple [**0**]; sclerite connecting base of valval costa to juxta wide, elbowed [**1**].
26. Membrane surrounding phallus base simple [**0**]; membrane surrounding phallus base bearing a small dorsal field of anteriorly-directed spines [**1**]; membrane surrounding phallus base with a large dorsal field of spines [**2**].
27. Spines surrounding phallus base short [**0**]; spines surrounding phallus base relatively long [**1**]; spines surrounding phallus base extremely robust, thorn-like [**2**]; two lengths of spines surrounding phallus base [**3**]; spines absent at phallus base (outgroup characteristic) [?].
28. A uniform field of spines surrounding phallus base [**0**]; spines in membrane surrounding phallus base arranged in a series of longitudinal rows [**1**]; spines surrounding phallus base arranged in two longitudinal rows [**2**]; a small group of lateral spines on either side of phallus, in addition to larger dorsal group [**3**]; spines absent at phallus base (outgroup characteristic) [?].
29. Region of manica dorsal to spine-field simple [**0**]; region of manica dorsal to spine-field bearing a thin, Y-shaped sclerite [**1**].
30. Juxta narrow at base [**0**]; juxta wide at base [**1**].
31. Outer surface of juxta slightly convex or flat [**0**]; juxta bearing a pair of lateral depressions [**1**].
32. Juxta gradually narrowing dorsally [**0**]; juxta abruptly narrowing in upper third [**1**].
33. Dorsal margin of juxta relatively wide, with a V-shaped or U-shaped mesal excavation [**0**]; dorsal margin of juxta narrow but truncate, with a small down-curved lip [**1**]; dorsal part of juxta acute [**2**]; dorsal margin of juxta forming a sclerotized, down-curved, horn-like structure [**3**].
34. Area between phallus and juxta simple [**0**]; a narrow, rod-like sclerite located between juxta and phallus [**1**].
35. Costa of valva narrow and band-like [**0**]; costa extremely narrow, rod-like [**1**]; costa relatively wide [**2**]; dorsal margin of valva membranous, costa apparently absent [**3**].
36. Costa long, extending nearly to valva apex [**0**]; costa somewhat shortened, falling well short of valva apex [**1**].
37. Dorsal margin of costa simple [**0**]; dorsal margin of costa bearing a short, elbow-like process near apex [**1**]; dorsal margin of costa bearing a pair of large thorn-like processes [**2**].
38. Valva relatively wide, dorsal and ventral margins roughly parallel [**0**]; valve wide, expanded toward apex [**1**]; valva extremely wide, forming a large oval [**2**]; valva abruptly narrowing toward apex [**3**].
39. Valva apex rounded [**0**]; valva apex slightly acute, triangular [**1**]; valva apex strongly acute, apex blade-like [**2**].
40. Inner surface of valva bearing setae for nearly its entire length [**0**]; inner surface of valva naked in basal half [**1**].
41. Apex of valva with fine, hair-like setae, similar to those covering remainder of valva [**0**]; valva with a brush of bristle-like or spine-like setae near apex, contrasting with remaining setae [**1**].
42. Inner surface of valva covered with hair-like setae only [**0**]; inner surface of valva covered with hair-like setae and pedicellate scales [**1**].
43. Valva without an isolated set of long setae near apex of sacculus [**0**]; ventral margin of valva with a secondary group of robust setae near apex of sacculus [**1**]; setae at apex of sacculus extremely long, longer than width of valva [**2**].
44. Valva lacking a row of setae along inner margin of sacculus [**0**]; valva with a group of long, hair-like setae along inner margin of sacculus [**1**]; valva with a dense group of bristle-like setae along inner margin of sacculus [**2**].
45. Sacculus heavily sclerotized [**0**]; sacculus lightly sclerotized [**1**]; area of sacculus membranous, sacculus apparently absent [**2**].
46. Sacculus approximately ½ as long as valva [**0**]; sacculus long, extending 2/3 or more the length of valva [**1**]; sacculus short, less than ⅓ as long as valva [**2**]; sacculus absent [?].
47. Sacculus relatively narrow [**0**]; sacculus wide [**1**]; sacculus an extremely broad, somewhat ovoid triangle [**2**]; sacculus apparently comprising a thin, sclerotized rod [**3**]; sacculus absent [?].
48. Ventral margin of sacculus roughly parallel to ventral margin of valva [**0**]; ventral margin of sacculus bowed outward [**1**]; ventral margin of sacculus with an elbow at base [**2**].
49. Ventral margin of valva smoothly contiguous with outer margin of sacculus [**0**]; a shallow excavation formed along ventral margin of valva near sacculus apex [**1**]; a pronounced notch formed along ventral margin of valva near sacculus apex [**2**].
50. Apex of sacculus simple [**0**]; sacculus bearing an apical spine [**1**]; sacculus bearing a transverse, apical flange [**2**]; sacculus with an acute, angled process at apex [**3**]; apex of sacculus bearing a large, spatulate process [**4**]; sacculus absent [?].
51. Phallus moderate in width, distal opening narrow [**0**]; phallus wide, distal opening wide [**1**]; phallus narrow [**2**].
52. Phallus moderate in length [**0**]; phallus elongate [**1**]; phallus short [**2**].
53. Base of phallus gradually narrowing anteriorly, rounded [**0**]; phallus base broadly rounded [**1**]; base of phallus narrow, somewhat horn-shaped [**2**].
54. Phallus base simple [**0**]; phallus base bearing a dorsal vertical flange [**1**].
55. Apex of phallus simple [**0**]; apex of phallus bearing a hook-like ventral process [**1**]; apex of phallus forming a large, blade-like ventral process [**2**]; apex of phallus bearing a prominent, spatulate ventral process [**3**]; apex of phallus bearing a narrow, acute ventral process [**4**].
56. Vesica comprising a single tube [**0**]; vesica bifurcate, comprising two appendices [**1**].
57. Vesica lacking spine-like cornuti [**0**]; vesica bearing a single distal group of one or more coarse, spine-like cornuti [**1**]; vesica with two distal groups of spine-like cornuti [**2**]; vesica with a single, tiny nub-like distal cornutus [**3**]; distal group of spine-like cornuti on vesica short, together forming a ratchet-like structure [**4**]; vesica bearing spine-like cornuti on dorsal appendix, and minute denticles on ventral appendix [**5**].
58. Main duct of vesica simple at base [**0**]; main duct of vesica minutely scobinate at base [**1**]; main duct of vesica with a patch of spines near base [**2**]; main duct of vesica with a thorn-like process at base [**3**].
59. Vesica lacking scobinate sclerites at base [**0**]; vesica with a pair of narrow, curved scobinate sclerites at base [**1**]; scobinate sclerites large [**2**]; vesica with a single, large scobinate sclerite at base [**3**].

Female-only characters

1. Antenna ciliate [**0**]; antenna bipectinate [**1**]; antennal cilia long and bristle-like [**2**].
2. Tergum 8 triangular, posterior margin gradually narrowed [**0**]; tergum 8 broadly triangular [**1**]; tergum 8 quadrate, posterior margin transverse [**2**]; tergum 8 a narrow, U-shaped band [**3**].
3. Ventral surface of A8 in area of postvaginal plate membranous or lightly sclerotized, PVP apparently absent [**0**]; postvaginal plate present, simple [**1**]; postvaginal plate large, somewhat quadrate [**2**]; postvaginal plate short, strap-like [**3**]; postvaginal plate convex [**4**]; postvaginal plate a triangle, tapering posteriorly [**5**].
4. Region of postvaginal plate smooth [**0**]; region of PVP bearing transverse striations [**1**].
5. Anterior apophyses shorter than posterior apophyses [**0**]; anterior apophyses elongate, equal in length to posterior apophyses [**1**].
6. Ostium large and funnel-shaped [**0**]; ostium forming a large, dorso-ventrally compressed, vase-like structure [**1**]; ostium comprising a narrow, transverse band [**2**]; ostium forming a large, concave structure, its dorsal wall striate [**3**]; region of ostium membranous [**4**].
7. Region between ostium and ductus bursae moderate in length [**0**]; region between ostium and DB long [**1**]; region between ostium and ductus bursae short [**2**]; no membranous region between ostium and ductus bursae [**3**].
8. Region between ostium and ductus bursae simple [**0**]; region between ostium and ductus bursae broadly membranous [**1**]; region between ostium and ductus bursae spiculate [**2**]; region between ostium and ductus bursae sclerotized [**3**]. region between ostium and ductus bursae absent [?].
9. Region between ostium and ductus bursae simple [**0**]; region between ostium and ductus bursae bearing a ventral appendix [**1**]; region between ostium and ductus bursae bearing a small knob-like ventral process [**2**]; region between ostium and ductus bursae absent [?].
10. Ductus bursae moderate in length [**0**]; ductus bursae short [**1**]; ductus bursae elongate [**2**]; ductus bursae apparently absent [**3**].
11. Ductus bursae relatively narrow [**0**]; ductus bursae wide [**1**]; ductus bursae extremely narrow [**2**]; ductus bursae absent [?].
12. Ductus bursae heavily sclerotized [**0**]; ductus bursae lightly sclerotized [**1**]; ductus bursae membranous [**2**]; ductus bursae absent [?].
13. Lateral margins of ductus bursae simple [**0**]; lateral margins of ductus bursae rolled upward, ductus U-shaped in cross section [**1**]; lateral margins of ductus bursae rolled strongly inward, meeting near mid-line [**2**]; ductus bursae absent [?].
14. Ductus seminalis arising from a small narrow appendix at base of CB [**0**]; DS arising from an elongate, triangular appendix at base of CB [**1**]; DS arising from a large, sac-like appendix at base of CB [**2**].
15. Ductus seminalis arising ventrally and curling to moth’s right [**0**]; ductus seminalis arising laterally on right side [**1**]; ductus seminalis arising dorsally and curling to the left [**2**]; DS arising laterally on left side and curling right [**3].**
16. Base of corpus bursae spineless [**0**]; base of corpus bursae bearing a group of internal spines immediately beyond ductus seminalis [**1**]; base of corpus bursae bearing an irregular, sclerotized plate [**2**].
17. Area of corpus bursae basal to signum mostly membranous [**0**]; area of CB basal to signum bearing longitudinal striae [**1**]; area of CB basal to signum sclerotized [**2**].
18. Base of corpus bursae rounded, contiguous with remainder of corpus [**0**]; base of corpus bursae forming a separate neck-like constriction [**1**].
19. Surface of corpus bursae lacking a covering of internal spicules (most of ingroup *Eois*) [**0**]; entire surface of corpus bursae with a dense covering of internal spicules (most of outgroup) [**1**]; basal 2/3 of corpus bursae covered with internal spicules [**2**].
20. Signum comprising an ovoid patch of short spines [**0**]; signum horn-like, its base partially protruding from CB [**1**]; horn seemingly reduced, barely projecting above surface of bursa [**2**]; signum absent [**3**].
21. Signum located ventrally [**0**]; signum located laterally on right side of CB [**1**]; signum located laterally on left side of CB [**2**]; signum located dorsally [**3**]; signum absent [?].
22. Internal part of horn-like signum narrow, curved, dentate along lateral margin [**0**]; internal part of horn-like signum wing-shaped, lateral margins serrate [**1**]; internal part of horn-like signum spatulate, lateral margins smooth [**2**]; internal part of horn-like signum forming a huge, claw-like structure [**3**]; internal part of horn-like signum comprised of long, curved spines [**4**]; internal part of horn-like signum smooth, horn-like [**5**]; horn-like signum absent [?].
23. Corpus bursae lacking a sclerotized crescent surrounding signum (both outgroup, most ingroup *Eois*) [**0**]; a sclerotized crescent, variable in size, present in CB membrane surrounding signum [**1**].
24. Corpus bursae without a sclerite arising from signum [**0**]; a spinose sclerite arising from signum, sclerite narrow, strap-like, wrapping around CB [**1**]; spinose sclerite becoming broad and plate-like [**2**]; signum sclerite a large ovoid plate, its outer margin bordered with long, curved spines [**3**]; signum sclerite reduced, thin, spines few or absent [**4**]; signum sclerite short, covered with a mass of fine internal bristles [**5**].
25. Central area of corpus bursae simple, without modifications beyond signum [**0**]; central area of CB with a large melanized area, covered in longitudinal striae [**1**]; area of CB beyond signum sclerite with an inset, well-defined rugose area [**2**]; central area of CB with a rugose, transverse fold [**3**]; corpus bursae with a smooth, inset sclerite in addition to signum sclerite [**4**]; central area of CB with a single, dentate pocket [**5**]; central area of CB bearing a pair of deep, spinose pockets [**6**]; central area of corpus bursae with a transverse sclerotized band [**7**]; central area of CB broadly sclerotized [**8**].
26. Corpus bursae single-parted, distal appendix absent [**0**]; CB composed of two parts, distal portion broadly attached to remainder of corpus [**1**]; CB composed of two parts, distal appendix with a relatively narrow, cylindrical attachment to remainder of corpus [**2**]; secondary appendix with a narrow, neck-like attachment to CB [**3**].
27. Distal appendix of corpus bursae smooth [**0**]; distal appendix of corpus bursae minutely wrinkled [**1**]; membrane of distal appendix delicate, fragile [**2**]; distal appendix absent [?].
28. Dorsal membrane between Tg8 and papillae anales simple, with a small membranous invagination [**0**]; membrane between Tg8 and papillae anales bearing a large dorsal sac [**1**].
29. Papillae anales roughly triangular in shape, distal portion rounded [**0**]; papillae anales elongate, distal portion rounded or acute [**1**]; papillae anales short, trapezoidal [**2**]; papillae anales sickle-shaped, acute at apex [**3**]; papillae anales extremely narrow [**4**].
30. Papillae anales lacking an apical hook [**0**]; papillae anales with a tiny apical hook [**1**].
31. Papillae anales evenly setose, bristles absent [**0**]; base of papillae anales with a series of longitudinal striae, a few scattered bristles present [**1**]; base of papillae anales heavily striate, with a dorsal corona of long, down-curved bristles [**2**].
32. Surface of papillae anales simple [**0**]; surface of papillae anales covered with a series of longitudinal striae [**1**].

**Table A1.** Character matrix of morphological character states of species *E.parumsimii* and *E.pseudolivacea* from a larger *Eois* data matrix.

Char. #	*E.parumsimii*	*E.pseudolivacea*	Char. #	*E.parumsimii*	*E.pseudolivacea*	Char. #	*E.parumsimii*	*E.pseudolivacea*
**1**	1	1	**41**	0	0	**81**	1	1
**2**	1	1	**42**	2	2	**82**	0	0
**3**	0	0	**43**	0	0	**83**	1	1
**4**	0	0	**44**	1	2	**84**	1	1
**5**	0	0	**45**	0	0	**85**	0	0
**6**	1	1	**46**	1	1	**86**	1	0
**7**	1	1	**47**	0	0	**87**	1	1
**8**	1	1	**48**	1	1	**88**	1	1
**9**	1	1	**49**	1	1	**89**	1	1
**10**	1	1	**50**	0	0	**90**	0	0
**11**	0	0	**51**	0	0	**91**	0	0
**12**	0	0	**52**	0	0	**92**	0	0
**13**	0	0	**53**	0	0	**93**	0	0
**14**	1	1	**54**	0	0	**94**	0	0
**15**	0	0	**55**	0	0	**95**	1	1
**16**	0	0	**56**	0	0	**96**	2	2
**17**	0	0	**57**	1	1	**97**	1	1
**18**	1	1	**58**	0	0	**98**	0	0
**19**	1	1	**59**	2	2	**99**	1	1
**20**	1	1	**60**	0	0	**100**	0	0
**21**	1	1	**61**	1	1	**101**	0	0
**22**	0	0	**62**	0	0	**102**	?	?
**23**	1	1	**63**	0	0	**103**	1	1
**24**	1	1	**64**	1	1	**104**	2	4
**25**	1	1	**65**	1	1	**105**	0	0
**26**	0	0	**66**	0	0	**106**	0	0
**27**	1	1	**67**	1	1	**107**	0	0
**28**	0	0	**68**	0	0			
**29**	0	0	**69**	2	2			
**30**	2	2	**70**	0	0			
**31**	1	1	**71**	2	2			
**32**	4	4	**72**	1	1			
**33**	1	1	**73**	1	2			
**34**	0	0	**74**	1	1			
**35**	0	0	**75**	3	3			
**36**	1	1	**76**	0	0			
**37**	3	2	**77**	2	2			
**38**	2	2	**78**	0	0			
**39**	0	0	**79**	1	1			
**40**	0	0	**80**	0	0			
